# Solid-Ejecta Morphology and Elemental Composition During Thermal Runaway of 58 Ah NCM811 Lithium-Ion Cells: Representative Near-Field Thermal-Flow Behavior

**DOI:** 10.3390/ma19194033

**Published:** 2026-09-22

**Authors:** Yunfei Lou, Ningning Wei

**Affiliations:** 1 Intelligent Manufacturing School, Jilin Tiedao University, Jilin 132299, China; 2 School of Energy and Mechanical Engineering, Dezhou University, Dezhou 253023, China

**Keywords:** lithium-ion battery, thermal runaway, solid ejecta, SEM/EDS characterization, reacting jet, thermal-flow behavior

## Abstract

Thermal runaway (TR) of lithium-ion batteries generates both gaseous and solid ejecta, yet the material characteristics of the expelled solids and their surrounding thermal-flow environment are often examined separately. In this study, solid ejecta recovered from three repeated thermal-runaway tests of 58 Ah Li(Ni_0.8_Co_0.1_Mn_0.1_)O_2_ (NCM811) cells under nitrogen were characterized using scanning electron microscopy (SEM) and energy-dispersive X-ray spectroscopy (EDS). A separate two-dimensional reacting-jet model was established to provide representative near-field thermal-flow context under a high-intensity ejection condition in ambient air. SEM observations at 500×–10,000× revealed irregular fragments, lamellar structures, agglomerates, and fine-particle deposits, indicating pronounced morphological heterogeneity of the recovered solids. In the local selected EDS region, Ni, Mn, and Co collectively accounted for 54.61 wt.% of the normalized elemental signal, together with substantial C, O, and F. The numerical model employed a reduced CO_2_-CO-H_2_-O_2_ mixture, a 12.5 mm vent inlet width, an inlet velocity of 90 m/s, and an inlet temperature of 941.2 °C. The maximum resolved velocity reached approximately 104 m/s near the vent, whereas the maximum simulated temperature was approximately 1806 °C, which occurred farther downstream. These results provide complementary insight into the post-failure material characteristics of solid ejecta and the representative near-field thermal-flow behavior associated with severe lithium-ion battery thermal runaway. The numerical results are intended as representative spatial context rather than a direct reconstruction of the nitrogen-atmosphere experiments.

## 1. Introduction

Thermal runaway (TR) of lithium-ion batteries (LIB) involves rapid heat generation, gas release, pressure rise, material ejection, and, under suitable conditions, ignition and flame development [1,2,3]. Considerable research has focused on cell-temperature evolution, vent-gas composition, pressure generation, fire behavior, and TR propagation between adjacent cells [4,5,6]. These studies have established the importance of gaseous products and flame development in determining the severity of battery failure. However, TR does not generate only gaseous products [7,8,9]. Solid and semi-solid materials can also be expelled from the cell at high velocity, particularly during intense venting and rupture events, introducing an additional transport pathway that remains less extensively characterized.

Our previous experiments using 58 Ah prismatic Li(Ni_0.8_Co_0.1_Mn_0.1_)O_2_ (NCM811) LIBs showed that the most intense stage of thermal runaway was accompanied by the persistent ejection of luminous solid particulates and sparks. The apparent ejecta velocity reached at least 90 m/s, and solid particulate material was observed throughout the intense ejection period [10]. These observations highlighted the importance of solid-material transport during severe thermal runaway. However, our previous work focused primarily on macroscopic ejection characteristics, including ejecta velocity, propagation behavior, mass loss, and flame development, and did not further characterize the microscopic morphology or elemental composition of the recovered solid ejecta. The present study therefore extends that work by combining direct SEM/EDS characterization of recovered particulate material with representative numerical analysis of the surrounding reacting-jet environment.

The gas-phase environment surrounding the ejecta is also highly dynamic. In our previous confined-space investigation of NCM811 thermal runaway under controlled oxygen concentrations, the major post-event gaseous products included H_2_, CO_2_, CO, CH_4_, and C_2_H_4_, and the subsequent combustion behavior was strongly influenced by the surrounding oxygen availability [11,12]. For the 0% O_2_ tests from which the solid ejecta examined in the present study were recovered, the first vent occurred at a cell-surface temperature of approximately 155 °C, followed by thermal-runaway onset at approximately 170 °C, while the peak measured vent-jet temperature reached approximately 353 °C [12]. However, the gas samples reported in that study represented post-event mixed chamber gas rather than the complete time-resolved composition of the transient hot vent jet. Consequently, those measurements provide useful experimental context but do not permit direct reconstruction of the instantaneous reacting-jet composition.

Solid-material ejection and gas-phase combustion are often investigated separately even though they occur simultaneously during severe TR [13,14,15]. From an engineering perspective, this distinction is important because expelled fragments and finer particulates are transported within a rapidly evolving gas-phase environment. Their motion and deposition are influenced by the surrounding velocity and temperature fields, while the gas phase governs turbulent mixing, heat release, and downstream thermal development [16,17,18]. A more integrated description of the near-field thermal-flow behavior therefore requires consideration of both the expelled solid phase and the surrounding gas-phase environment through which it is transported.

Scanning electron microscopy (SEM) can reveal whether collected ejecta consist primarily of fragmented structures, lamellar debris, or agglomerated fine material, while energy-dispersive X-ray spectroscopy (EDS) provides information on the elements present within selected regions [19,20,21]. These methods do not reconstruct the transient thermal-flow environment surrounding the ejecta. Conversely, numerical simulation can resolve representative spatial distributions of velocity, temperature, and combustion products, but its interpretation depends on uncertain transient vent velocity and gas chemistry [22,23,24]. Using the two approaches together therefore places solid-phase characterization alongside representative gas-phase conditions without requiring a one-to-one reconstruction of the same test atmosphere.

The overall framework of the present study is shown in Figure 1. Solid ejecta recovered from three repeated nitrogen-atmosphere thermal-runaway tests were pooled into a composite sample for multiscale SEM and local EDS characterization. Separately, a representative high-intensity reacting jet into ambient air was simulated using a reduced CO_2_-CO-H_2_-O_2_ mixture and inlet conditions referenced to previously measured high-intensity ejection behavior. The two analyses are complementary rather than a one-to-one reconstruction of the same atmospheric condition. The present study therefore aims to characterize the morphology and local elemental composition of the recovered solid ejecta and to examine the representative near-field thermal-flow behavior of a high-intensity reacting jet, with particular emphasis on the spatial development of velocity, temperature, and combustion-product transport.

## 2. Materials and Methods

### 2.1. Battery Cells, Solid-Ejecta Sampling, and SEM/EDS Characterization

Commercial 58 Ah prismatic lithium-ion cells (CALB Group Co., Ltd., Changzhou, China) employing an NCM811 cathode and graphite anode were used. The cells had a nominal mass of 926 ± 15 g and dimensions of approximately 148.24 × 26.66 × 102.8 mm. Aluminum and copper foils were used as the cathode and anode current collectors, respectively, and the cell casing was aluminum alloy.

The solid ejecta examined in the present study originated from three repeated 0% O_2_ thermal-runaway tests conducted under nitrogen using the same 58 Ah NCM811 cell type and manufacturing batch as in our previous confined-space study [12]. The tests were performed in the 250 L sealed heating and pressure chamber described previously. Thermal abuse was imposed using the heating–waiting–seeking (HWS) protocol reported in Ref. [12]. During the heating stage, the programmed temperature was increased in 5 K increments, and the cell was maintained at each temperature level for 30 min to reduce transient thermal gradients and allow thermal stabilization. In these tests, the first vent occurred at a cell-surface temperature of approximately 155 °C, followed by thermal-runaway onset at approximately 170 °C. Under the 0% O_2_ condition, the peak measured vent-jet temperature was approximately 353 °C [12]. These temperature values characterize the thermal conditions of the parent thermal-runaway experiments rather than the temperature of the recovered particulate material at the time of sampling. After each experiment had fully cooled, settleable solid ejecta deposited on the bottom of the sealed chamber were collected using a brush and sealed in medical glass vials for storage. Equal masses of recovered particulate material from each of the three independent 0% O_2_ tests were then combined to form the composite sample used for SEM and EDS characterization. Accordingly, the present analysis describes the representative morphology and local elemental composition of an equal-mass pooled sample rather than test-by-test variability. The principal battery, characterization, and numerical-model parameters are summarized in Table 1.

The composite particulate sample was prepared for microscopy by trained facility personnel. A small amount of powder was transferred onto the microscopy specimen mount before analysis. Detailed records of the mounting medium and any conductive-coating step were not available for retrospective reporting. The ejecta were examined using a Merlin Compact field-emission scanning electron microscope (Carl Zeiss Microscopy GmbH, Jena, Germany) equipped with an EDS system. Representative SEM images were acquired at magnifications of 500×, 1000×, 2000×, and 10,000× to characterize morphology over multiple length scales. The SEM observations were used primarily for qualitative assessments of fragmentation, agglomeration, lamellar features, surface roughness, and fine-particle deposition. Because the analyzed fields represented selected microscopic regions rather than statistically randomized bulk sampling, no bulk particle-size distribution was inferred.

Local elemental characterization was subsequently conducted using the EDS system on a representative particulate region selected from the composite ejecta sample. Only one microscopic region of the pooled composite sample was quantitatively analyzed by EDS in the present study. Because equal masses from the three independent tests had been combined before microscopic characterization, test-resolved elemental variability could not be determined. In addition, the EDS region was not selected through a statistically randomized spatial-sampling procedure; therefore, the reported elemental fractions should be interpreted as local compositional information rather than as a statistically representative average of the recovered ejecta. The spectrum was acquired at an accelerating voltage of 15 kV and a live acquisition time of approximately 60 s. The original spectrum contained signals corresponding to C, O, F, Al, Si, S, Mn, Co, and Ni. Lithium was not quantified by the present EDS analysis.

EDS concentrations were normalized to the analyzed elemental signal. Because this measurement represented a selected local particulate region, the resulting elemental fractions were interpreted as local characteristics rather than as the bulk-average composition of the entire collected ejecta sample.

### 2.2. Reduced Representation of the Vent-Gas Mixture

The reduced mixture was derived from the experimentally characterized post-event gas composition reported in our previous study [12] using an elemental-equivalent reduction procedure. Carbon-, hydrogen-, and oxygen-containing species in the original mixture were represented by a reduced set of species while preserving the overall elemental molar balance. CO and H_2_ were retained as the principal combustible surrogate species, CO_2_ as the major carbon-containing diluent, and the residual O_2_ fraction was preserved. The resulting reduced model input consisted of 31.75 vol.% CO_2_, 35.82 vol.% CO, 32.06 vol.% H_2_, and 0.37 vol.% O_2_. Under ideal-gas conditions, the volume fractions were treated as equivalent to molar fractions. Because the original measurements represented post-event mixed chamber gas rather than the instantaneous hot vent plume, the reduced composition was used as a representative model input rather than a time-resolved vent composition. Accordingly, the adopted reduced chemistry defines a representative reacting-jet scenario and should not be interpreted as a reconstruction of the instantaneous chemical composition or detailed reaction kinetics of the actual vent plume.

Within the combustible fraction of the reduced mixture, CO and H_2_ accounted for 52.77% and 47.23%, respectively. The principal global oxidation reactions are given in Equations (1) and (2):

(1) $$CO+0.5\;O_{2}\to CO_{2}$$

(2) $$H_{2}+0.5\;O_{2}\to H_{2}O$$

Accordingly, the normalized combustible mixture is given in Equation (3):

(3) $$0.5277\;CO+0.4723\;H_{2}+0.5\;O_{2}\to 0.5277\;CO_{2}+0.4723\;H_{2}O$$

Mass transfer in the reacting jet was modeled by solving for the mass fractions of six species: CO, H_2_, O_2_, CO_2_, H_2_O, and N_2_. To account for turbulence in the flow-field solution, the *k*–*ω* turbulence model was employed. When a turbulence model is used in the Reacting Flow interface, the production rate of species *i* due to reaction *j* (SI unit: kg/(m^3^·s)) is taken as the smaller of the mean-field closure reaction rate and the eddy-dissipation-model reaction rate, as given in Equation (4) [25]:

(4) $$R_{ij}=v_{ij}M_{i}\cdot \left[r_{\mathrm{MVC},j},\;r_{\mathrm{ED},j}\right]$$

The mean-field closure reaction rate, given in Equation (5), is the kinetic reaction rate expressed in terms of the mean mass fractions. It represents the characteristic reaction rate for reactions whose chemical kinetics are slower than turbulent mixing, as well as for regions where the effects of turbulence are negligible. The applicability of this reaction-rate formulation can be quantified using the Damköhler number, defined as the ratio of the turbulent time scale ($\tau_{T}$) to the chemical reaction time scale ($\tau_{c}$). The mean-field closure is applicable at low Damköhler numbers:

(5) $$D_{a}=\frac{\tau_{T}}{\tau_{c}}\ll 1$$

The reaction rate defined by the eddy-dissipation model is given in Equation (6):

(6) $$r_{\mathrm{ED},j}=\frac{\alpha_{j}}{\tau_{T}}\rho \cdot \min\left[\min(\frac{\omega_{r}}{\nu_{rj}},\beta \sum_{p}\left(\frac{\omega_{p}}{\nu_{pj}M_{p}}\right)\right]$$

where $\tau_{T}$ (SI unit: s) is the turbulent mixing time scale, *ρ* is the mixture density (SI unit: kg/m^3^), ω is the species mass fraction, ν denotes the stoichiometric coefficient, and M is the molar mass (SI unit: kg/mol). The properties of the reactants involved in the reaction are denoted by the subscript “r”, whereas those of the products are denoted by the subscript “*p*”.

For 100 mol of the adopted reduced vent-gas mixture, complete oxidation of the CO and H_2_ fractions requires approximately 33.94 mol O_2_, whereas the inlet mixture itself contains only 0.37 mol O_2_. Therefore, combustion under the modeled condition depends predominantly on entrainment and turbulent mixing with ambient air.

Reaction heat and heat capacity: The present model includes a thermodynamic system containing all species considered in the simulation, which is used to define the temperature- and composition-dependent properties of individual species and the gas mixture [25]. After coupling the chemistry interface with the thermodynamic system, the required thermodynamic and transport properties are automatically defined. The two key thermal properties considered are the heat of reaction and the heat capacity of the mixture. Figure 2a shows the variation in the enthalpy of formation of each individual species with temperature. The enthalpy of formation increases with increasing temperature for all species, indicating that temperature dependence should be taken into account to improve the accuracy of the calculation. The enthalpy of formation and heat-capacity data were obtained from the built-in thermodynamic database of COMSOL Multiphysics^®^ 6.2. Figure 2b presents the temperature dependence of the heat capacity of all species included in the system. Similar to the enthalpy of formation, the heat capacity of each species increases with temperature. Therefore, the temperature dependence of heat capacity was also incorporated into the model.

The reduced representation was adopted to capture the principal heat-release, flow, and combustion-product transport characteristics of the representative high-intensity ejection case. It was not intended to reproduce the complete transient chemical kinetics of the original multicomponent thermal-runaway vent gas.

### 2.3. Representative Near-Field Thermal-Flow Model

The ambient-air reacting-jet simulation was intentionally treated as a separate representative scenario and was not intended to reproduce the nitrogen-atmosphere experiments from which the solid ejecta were recovered. A two-dimensional numerical model was established using COMSOL Multiphysics 6.2 to investigate the near-field thermal-flow behavior of a representative high-intensity thermal-runaway reacting jet. Post-test inspection indicated that the ruptured safety-valve opening was approximately circular, with an observed opening width of about 12.5 mm. In the present two-dimensional representation, this value was prescribed as the inlet width of the vent opening rather than as a three-dimensional area-equivalent circular vent. The surrounding environment was set to 25 °C and standard atmospheric pressure (101.325 kPa), with a coflow air velocity of 0.2 m/s.

A representative inlet velocity of 90 m/s was prescribed based on the maximum apparent ejecta velocity reported in our previous thermal-runaway experiments [10]. The inlet temperature was set to 941.2 °C using the previously measured peak jet-zone temperature, *T*_E_, as a boundary-condition proxy [10]. Because *T*_E_ was measured approximately 1 cm above the safety-valve outlet, it does not represent a direct measurement of the instantaneous vent-plane gas temperature. Similarly, the 90 m/s value was derived from the apparent motion of luminous particulate ejecta rather than direct bulk-gas velocimetry. The numerical case is therefore interpreted as a representative high-intensity ejection condition rather than an exact reconstruction of the transient venting process. The prescribed inlet velocity of 90 m/s and temperature of 941.2 °C were derived from prior experimental observations and used as scenario-defining boundary conditions rather than direct measurements of the bulk gas velocity or vent-plane gas temperature. Accordingly, the velocity and temperature fields reported below represent numerical predictions for the prescribed reacting-jet scenario. Because no independent gas-phase velocimetry, vent-plane thermometry, spatial temperature-field measurements, or parametric sensitivity analysis were available, the model predictions could not be directly validated, and the influence of boundary-condition uncertainty on the predicted peak values and their spatial locations was not quantified. The resulting numerical fields should therefore be interpreted as scenario-dependent predictions rather than uniquely determined reconstructions of the actual venting process.

At the safety-valve outlet, the reduced fuel mixture and coflow air formed an unconfined turbulent jet. The fuel stream was ignited at the valve outlet and treated as a non-premixed turbulent reacting jet. Ambient oxidizer was supplied by jet entrainment, free spreading, and turbulent mixing with the surrounding air, allowing local, near-stoichiometric fuel-oxidizer conditions to develop in the reaction zone.

A k-ω turbulence model was employed to describe the turbulent flow field. The combustion process was represented using the two global oxidation reactions given in Equations (1) and (2), together with an eddy-dissipation treatment. Under the adopted fast-chemistry assumption, the effective reaction rate was predominantly controlled by turbulent mixing. The eddy-dissipation parameters were set to *α* = 4 and *β* = 0.5.

The modeled reacting system included CO, H_2_, O_2_, CO_2_, H_2_O, and N_2_. Temperature-dependent thermodynamic properties, including species enthalpies and heat capacities, were evaluated using the thermodynamic property database incorporated in COMSOL Multiphysics 6.2. A stationary solution was obtained to characterize the spatial thermal-flow structure under the prescribed high-intensity boundary conditions. The model was therefore intended to represent a quasi-steady high-intensity ejection condition rather than the temporal evolution of the complete thermal-runaway venting process. Radiative heat transfer was enabled in the thermal model, while gravity was neglected and buoyancy-driven flow was therefore not considered. The outer boundaries of the external computational domain were assigned the COMSOL Open Boundary condition.

The computational domain was discretized using a finite-element mesh containing 13,101 vertices and 12,800 elements. The minimum and average element qualities were 0.3197 and 0.8019, respectively, and the modeled area was approximately 0.1624 m^2^. These values are reported to document the spatial discretization used for the representative numerical case. A formal grid-convergence or computational-domain sensitivity analysis was not performed for the present representative case. Accordingly, the mesh and domain parameters reported here document the numerical configuration used rather than establish mesh-independent or domain-independent predictions. The numerical results should therefore be interpreted within the limitations of this single stationary two-dimensional configuration. Because the present model was intended to provide representative thermal-flow context rather than a quantitatively validated reconstruction of the transient venting process, no formal mesh-independence analysis was performed. The numerical solution was post-processed to obtain the spatial distributions of velocity magnitude, temperature, and CO_2_ mass fraction. Centerline profiles were additionally extracted to quantify their downstream evolution.

### 2.4. Use of Generative Artificial Intelligence

Generative artificial intelligence was used only to assist with manuscript language refinement and the initial preparation of schematic illustrations. ChatGPT (GPT-5.6 Sol, OpenAI, San Francisco, CA, USA) was used for English-language editing to improve grammar, clarity, concision, and readability. OpenAI’s ChatGPT image-generation capability was used to assist in generating preliminary graphical layouts and selected illustrative elements for Figure 1 based on instructions provided by the authors. The AI-assisted figures were subsequently manually revised by the authors, including modifications to the text, labels, annotations, layout, and selected graphical elements. All scientific concepts, interpretations, annotations, and relationships presented in the final figures were determined and verified by the authors. Generative AI was not used to generate, modify, analyze, or interpret any experimental data, SEM/EDS results, numerical simulation results, quantitative values, or scientific conclusions.

## 3. Results and Discussion

### 3.1. Morphology and Elemental Characteristics of the Solid Ejecta

Figure 3 presents the SEM morphology of the collected solid ejecta over magnifications from 500× to 10,000×. Across all examined length scales, the material exhibited pronounced morphological heterogeneity rather than a uniform particulate structure.

At 500× magnification (Figure 3a), the field contained irregular coarse agglomerates, fragmented plate-like structures, and numerous finer particles distributed around and attached to larger fragments. The coexistence of these morphologies suggests that the ejecta consisted of multiple structural components rather than a homogeneous population of particles.

At 1000× magnification (Figure 3b), the hierarchical character became more evident. Larger structures displayed plate-like or lamellar features with fractured edges, whereas other regions comprised granular agglomerates formed from much finer particles. Fine particulate material was also attached to the surfaces of larger fragments.

At 2000× magnification (Figure 3c), partially separated layers, irregular fracture surfaces, and extensive fine-particle deposition were more clearly resolved. These structures are compatible with fragmentation and disruption of layered cell components during the rapid gas-generation and ejection process, although the identity and formation history of individual structures cannot be assigned from morphology alone.

The 10,000× image (Figure 3d) further revealed stacked lamellar structures, interlayer gaps, fractured edges, and fine particles deposited on rough surfaces. No dominant population of smooth spherical particles was evident within the selected fields. The present SEM observations therefore provide stronger support for fragmentation, delamination, agglomeration, and particulate deposition than for extensive droplet-like melting and resolidification.

These microscopic observations complement the previously reported macroscopic behavior, in which luminous particulates persisted throughout intense ejection [10]. The present results extend those observations by demonstrating that the deposited solids retain complex multiscale structures after the event.

Figure 4 presents the selected region for EDS analysis and the corresponding elemental spectrum, while the quantitative elemental composition is summarized in Table 2. Nine elements, namely C, O, F, Al, Si, S, Mn, Co, and Ni, were detected within the analyzed particulate region, indicating a chemically heterogeneous composition. Among the transition metals, Ni, Mn, and Co were the predominant species, with Ni showing the highest relative contribution. The concurrent detection of nonmetallic and metallic elements further confirms that the solid ejecta consisted of multiple material components rather than a single homogeneous phase. These results are consistent with the heterogeneous morphology observed in the SEM images and provide additional compositional evidence for the complex nature of the thermal-runaway ejecta.

Ni, Mn, and Co collectively accounted for 54.61 wt.% of the normalized elemental signal, supporting a substantial cathode-derived contribution within the selected region. Importantly, these EDS-derived weight percentages describe only the normalized elemental composition within the selected microscopic analysis region. They do not quantify the total mass of recovered solid ejecta, the fraction of the original cell mass that was ejected, or the bulk-average composition of all ejecta. The local Ni-Mn-Co proportions should not be interpreted as pristine NCM811 stoichiometry because the measurement represents a heterogeneous particulate region rather than isolated cathode material.

The local transition-metal distribution also differs from the previously reported particle compositions for NMC811 TR. A previous NMC811 particle study [26], as summarized by Li et al. [27], reported a combined Ni, Mn, and Co mass fraction of 77.59 wt.%, with the relative transition-metal proportions remaining close to the nominal cathode stoichiometry. In comparison, Ni, Mn, and Co accounted for 54.61 wt.% in the selected region examined here. Within the Ni-Mn-Co subset, the local atomic proportions were approximately 55.7:31.2:13.2, which differ from the nominal NCM811 ratio of 80:10:10. This difference should not be interpreted as a change in intrinsic cathode stoichiometry; rather, it is consistent with local compositional heterogeneity in the analyzed particulate region, which was not equivalent to isolated pristine cathode material.

Direct quantitative comparison across studies nevertheless requires caution. Reported elemental fractions can depend on the sampled particle-size range, collection location and atmosphere, whether deposited solids or airborne aerosols are analyzed, the analytical technique, and the normalization basis used to report composition [27]. The present result represents one local EDS region within a pooled sample recovered from three nitrogen-atmosphere tests and therefore should not be treated as the bulk-average composition of all NCM811 thermal-runaway particles. The cross-study difference is more appropriately interpreted as evidence of sampling and region-dependent compositional variability than as a contradiction between datasets.

Selected literature values are summarized in Table 3 to illustrate the range of reported elemental compositions and the importance of sampling and analytical basis when interpreting cross-study differences.

Carbon accounted for 15.49 wt.% and may originate from graphite, conductive carbon, carbonized organic components, or a combination of these sources. Because detailed records of the specimen mounting medium were unavailable, a contribution from sample preparation cannot be completely excluded. The carbon signal was therefore interpreted conservatively and was not assigned uniquely to a specific battery component.

Fluorine accounted for 9.85 wt.% and may reflect fluorine-containing residues associated with electrolyte-salt decomposition and/or fluorinated binder components. EDS, however, establishes only the presence of elemental F and does not identify its chemical state. Specific compounds such as LiF therefore cannot be confirmed from the present measurement.

Oxygen represented 18.51 wt.% of the analyzed region and was detected together with the transition-metal-rich fraction. This is consistent with oxygen-containing electrode material and/or thermally transformed residues. The minor Al content may originate from the cathode current collector or other aluminum-containing cell structures. Si and S were present only at trace concentrations.

Taken together, the SEM and EDS results indicate pronounced morphological heterogeneity and a locally multicomponent elemental composition in the recovered ejecta. The results indicate that thermal-runaway ejection can transport electrode-derived material and other internal cell components in addition to gaseous products and carbonaceous smoke.

From an engineering perspective, deposition of such particulate material on nearby cells, busbars, connectors, electronic components, or insulating surfaces could represent an additional contamination pathway. The present study did not directly measure electrical conductivity, insulation degradation, deposition efficiency, or secondary ignition probability; these therefore remain potential implications rather than demonstrated failure mechanisms.

Accordingly, the present EDS result should be regarded as a local compositional observation of the pooled sample rather than a measure of test-to-test or spatial variability.

### 3.2. Representative Near-Field Thermal-Flow and CO_2_ Transport Characteristics

Figure 5 presents the velocity, temperature, and CO_2_ mass-fraction fields together with centerline velocity and temperature profiles for the representative high-intensity case. Because the 90 m/s inlet velocity was referenced to apparent particulate-ejecta motion rather than direct bulk-gas velocimetry, the numerical results are interpreted as representative thermal-flow behavior of a high-intensity reacting jet rather than as an exact reconstruction of the transient venting process [25].

The velocity field in Figure 5a shows a narrow high-speed region immediately downstream of the vent, followed by rapid jet spreading and velocity decay. The maximum resolved velocity in the external flow field was approximately 104 m/s on the centerline at *y* ≈ 0.016 m, exceeding the prescribed inlet velocity of 90 m/s by 13.95 m/s, or approximately 15.5%. The local increase in velocity is consistent with thermal expansion and development of the reacting jet. As the gas temperature rises, the associated decrease in density and volumetric expansion can contribute to local flow acceleration. However, because an otherwise identical non-reacting case was not simulated, the increase above the prescribed inlet velocity cannot be attributed exclusively or quantitatively to chemical heat release. It is therefore interpreted as the combined response of the reacting, expanding, and developing jet.

Farther downstream, jet spreading, turbulent entrainment, and shear-layer mixing became increasingly important, resulting in substantial velocity decay. At *y* = 0.202 m, the simulated centerline velocity had decreased to approximately 34.6 m/s despite the development of the high-temperature reaction zone. Thus, although thermal expansion can contribute to local acceleration, it does not offset the overall downstream decay of axial velocity associated with free-jet development and entrainment.

In contrast to the near-vent velocity maximum, the maximum temperature occurred substantially farther downstream. The simulated temperature reached approximately 1806 °C at *y* ≈ 0.207 m near the centerline. As schematically illustrated in Figure 6, this downstream temperature maximum is consistent with the fuel-rich nature of the discharged mixture: after ejection, progressive entrainment of ambient air and turbulent mixing supply the oxygen required for oxidation, allowing stronger heat release to develop away from the vent. Similar coupling among vent-gas discharge, ambient-air entrainment, turbulent mixing, and downstream thermal development has been reported in previous numerical studies of LIB thermal runaway [29,30,31,32]. The predicted peak temperature and the downstream location of the high-temperature region are model-dependent and reflect the adopted reduced chemistry, thermodynamic properties, turbulent-mixing treatment, and prescribed boundary conditions. These quantities should therefore be interpreted as scenario-dependent numerical predictions rather than direct measurements or unique reconstructions of the transient vent plume. Accordingly, emphasis is placed on the representative spatial development of the velocity and temperature fields, particularly the separation between the near-vent high-velocity region and the downstream high-temperature region, rather than on treating the absolute peak values as experimentally validated quantities.

The velocity and temperature maxima were separated by approximately 0.19 m. This spatial offset indicates that the region of maximum gas velocity did not coincide with that of maximum thermal intensity. Under the representative modeled condition, the near field was characterized by high jet velocity together with local expansion of the reacting gas, whereas farther downstream the jet progressively entrained and mixed with ambient air, promoting stronger combustion and heat release even as the axial velocity continued to decrease. The resulting flow therefore exhibited a distinct near-field high-velocity region and a downstream high-temperature region.

The CO_2_ mass-fraction field provides complementary information on reaction-product formation, transport, and dilution. At the upstream centerline of the external flow field, the CO_2_ mass fraction was approximately 0.564. It increased slightly to approximately 0.577 at *y* ≈ 0.050 m before decreasing to approximately 0.388 near the axial location of the maximum-temperature region. The initial increase is consistent with CO oxidation contributing additional CO_2_ within the developing reaction zone, whereas the subsequent decrease reflects the progressively stronger influence of ambient-air entrainment and dilution as the jet spreads downstream.

The centerline profiles in Figure 5d further demonstrate this spatial evolution. The velocity reached its maximum close to the vent and subsequently decreased, whereas the temperature continued to rise downstream before reaching its maximum near *y* ≈ 0.207 m. These profiles demonstrate that peak flow velocity and peak thermal exposure were spatially separated under the representative reacting-jet condition rather than evolving synchronously along the jet axis.

These numerical observations provide gas-phase environmental context for the solid-ejecta characterization presented in Section 3.1. SEM and EDS characterize the morphology and local elemental composition of the recovered solid fraction, whereas the numerical model illustrates the surrounding high-velocity and high-temperature gas-phase environment under a representative peak-ejection condition. Particle trajectories, particle heating, deposition, and two-way gas–particle interactions were not explicitly modeled. Accordingly, no direct causal relationship is inferred between individual structures observed by SEM and specific locations or conditions within the simulated reacting field.

### 3.3. Thermal-Flow Interpretation and Limitations

The combined results demonstrate that the near-field thermal-flow behavior of severe thermal runaway is spatially nonuniform. The recovered ejecta contained fragmented and agglomerated solids with a locally multicomponent elemental composition, while the representative reacting-jet model revealed distinct high-velocity and high-temperature regions. These observations provide complementary information on the solid products and the surrounding thermal-flow environment during high-intensity ejection.

The SEM observations revealed the coexistence of coarser fragments and finer particulate material. Although differences in particle size and morphology may influence their post-ejection behavior, particle trajectories, deposition efficiency, and gas–particle interactions were not directly measured or modeled in the present study. Accordingly, no quantitative conclusions regarding particle transport or deposition are drawn, and any potential differences between coarse and fine ejecta are considered only as qualitative engineering implications.

The spatial separation of the velocity and temperature maxima further suggests that components close to the vent may experience stronger high-velocity flow exposure, whereas components farther downstream may encounter substantially lower axial velocity but greater thermal exposure. In the representative reacting-jet case, the external-flow velocity increased locally above the prescribed 90 m/s inlet value before subsequently decaying downstream, consistent with the combined effects of jet development and thermally induced gas expansion. The maximum temperature occurred much farther downstream after progressive entrainment and mixing with ambient air. Consequently, a single distance or scalar parameter may be insufficient to characterize the spatially varying thermal-flow behavior of the near-field reacting jet.

Several limitations bound this interpretation. First, particulate material from three nitrogen-atmosphere tests was combined on an equal-mass basis before microscopy. Consequently, the SEM images represent selected fields of view of the pooled material, and the EDS result represents one local microscopic region; neither test-to-test elemental variability nor spatial compositional variability can therefore be quantified from the present dataset. Second, EDS does not identify chemical phase, bonding state, or oxidation state. In addition, detailed records of the SEM mounting medium and any conductive-coating step were unavailable, so the carbon signal was interpreted conservatively. Third, the solid sample was recovered under nitrogen, whereas the numerical case represents a separate reacting jet into ambient air; the two analyses are complementary rather than a reconstruction of the same external atmosphere. Fourth, only one representative numerical condition was considered, and the 90 m/s inlet velocity was referenced to apparent particulate motion rather than direct bulk-gas velocimetry. The predicted local acceleration above the inlet velocity is physically consistent with expansion of the reacting heated gas, but its magnitude cannot be attributed exclusively to combustion because an otherwise identical non-reacting reference case was not simulated. Fifth, the reduced chemistry was derived from post-event mixed chamber gas rather than time-resolved vent-gas measurements and was intended to capture representative thermal-flow behavior rather than detailed transient chemistry. Consequently, both the simulated peak temperature and the spatial location of the high-temperature reaction region are scenario-dependent and should not be interpreted as unique predictions of the actual transient venting process. The post-rupture vent opening was represented in the 2D model by a 12.5 mm inlet width based on the observed opening width. The stationary model neglected gravity and buoyancy, so the predicted field emphasizes jet flow, turbulent mixing, chemical heat release, and radiative heat transfer under the prescribed boundary conditions. A formal grid-convergence and computational-domain sensitivity analysis was not conducted for the present representative numerical case. In addition, only one stationary two-dimensional configuration was evaluated. Consequently, the predicted peak values and their spatial locations have not been demonstrated to be independent of mesh resolution, domain extent, or dimensional/model-form assumptions. The numerical results are therefore interpreted as representative scenario-dependent fields rather than quantitatively verified predictions. Particle transport and gas–particle interactions were not modeled.

Despite these limitations, the combined approach extends prior macroscopic ejection observations by adding solid-ejecta morphology and local elemental characterization together with representative near-field thermal-flow context. The results highlight the spatially distinct development of high-velocity flow, downstream thermal intensity, and transported solid ejecta within the representative near-field environment.

## 4. Conclusions

This study characterized a composite solid-ejecta sample pooled from three repeated nitrogen-atmosphere thermal-runaway tests of 58 Ah NCM811 lithium-ion cells and complemented the experimental characterization with a separate representative reacting-jet simulation in ambient air.

SEM observations from 500× to 10,000× revealed irregular fragments, lamellar structures, agglomerates, interlayer separation, and fine-particle deposits, demonstrating pronounced multiscale morphological heterogeneity of the recovered solid ejecta. In the selected local EDS region, Ni, Mn, and Co collectively accounted for 54.61 wt.% of the normalized elemental signal, together with substantial C, O, and F. These results indicate a strong local contribution from cathode-derived materials; however, the reported EDS-derived weight percentages characterize only the normalized elemental composition within the selected microscopic region. They should not be interpreted as the bulk composition of the recovered ejecta, the mass fraction of the total recovered solid ejecta, the fraction of the original cell mass that was ejected, or as evidence for specific chemical phases.

For the representative numerical condition, the predicted external-flow velocity reached approximately 104 m/s near *y* ≈ 0.016 m, whereas the maximum simulated temperature of approximately 1806 °C occurred farther downstream near *y* ≈ 0.207 m. The approximately 0.19 m separation between the two maxima indicates that the high-velocity and high-temperature regions developed at distinctly different downstream locations. The local increase in velocity above the prescribed 90 m/s inlet value was consistent with the development and thermal expansion of the reacting jet, but its magnitude cannot be attributed exclusively to combustion in the absence of a corresponding non-reacting reference case.

Overall, the experimental and numerical results provide complementary information on the post-failure material characteristics of solid ejecta and the near-field thermal-flow environment associated with severe lithium-ion battery thermal runaway. The numerical simulation is intended to provide representative spatial context for a high-intensity ejection condition rather than a validated reconstruction of the nitrogen-atmosphere experiments. Future work should incorporate test-resolved particulate characterization, phase-sensitive chemical analysis, time-resolved vent-gas measurements, non-reacting comparison cases, and multiphase particle-transport modeling.

## Figures and Tables

**Figure 1:**
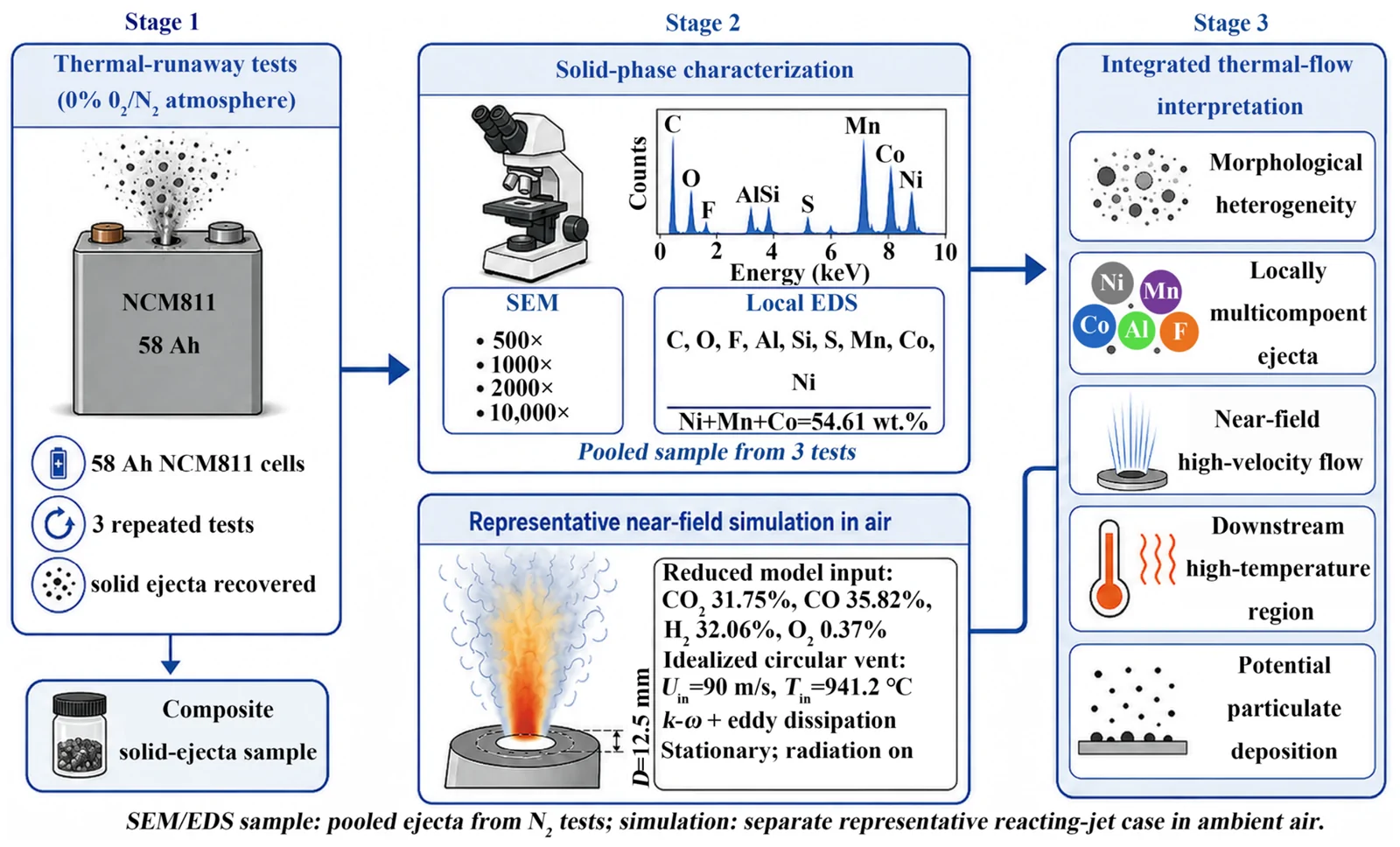
Overall study framework for post-test solid-ejecta characterization and representative reacting-jet simulation.

**Figure 2:**
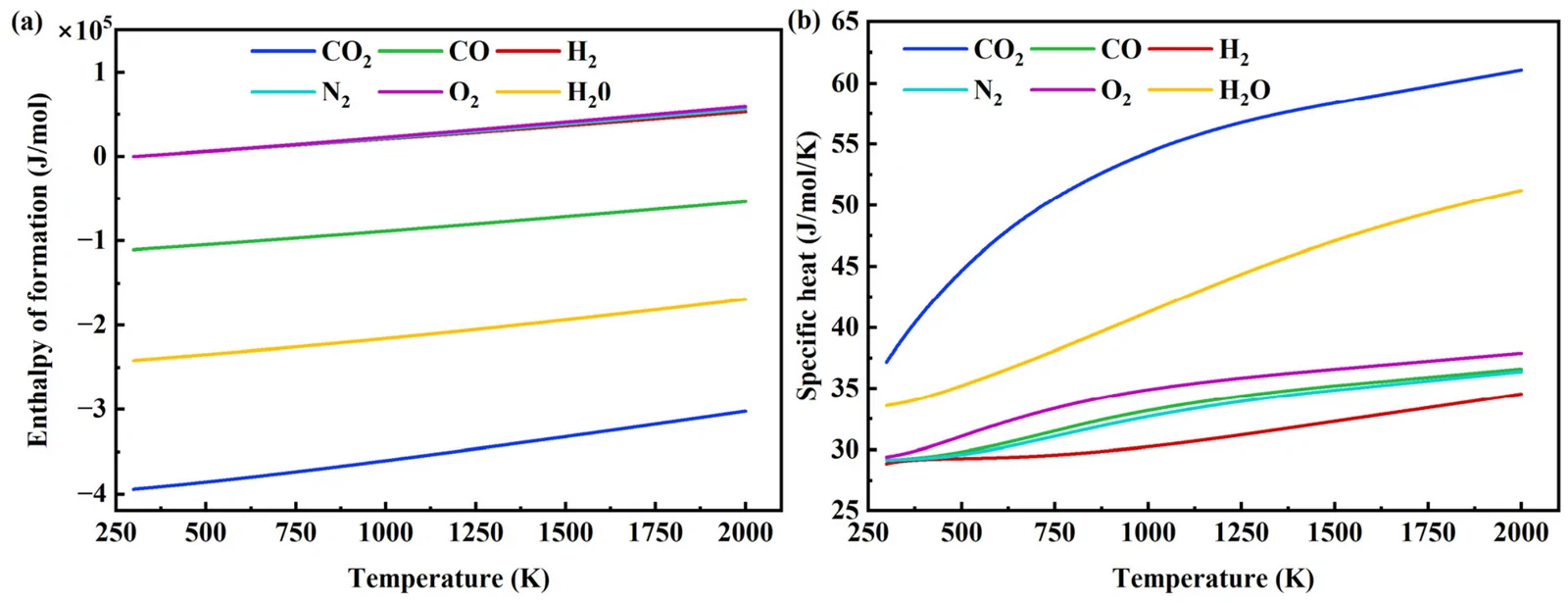
Temperature dependence of (**a**) species enthalpy of formation and (**b**) molar heat capacity used in the thermodynamic model.

**Figure 3:**
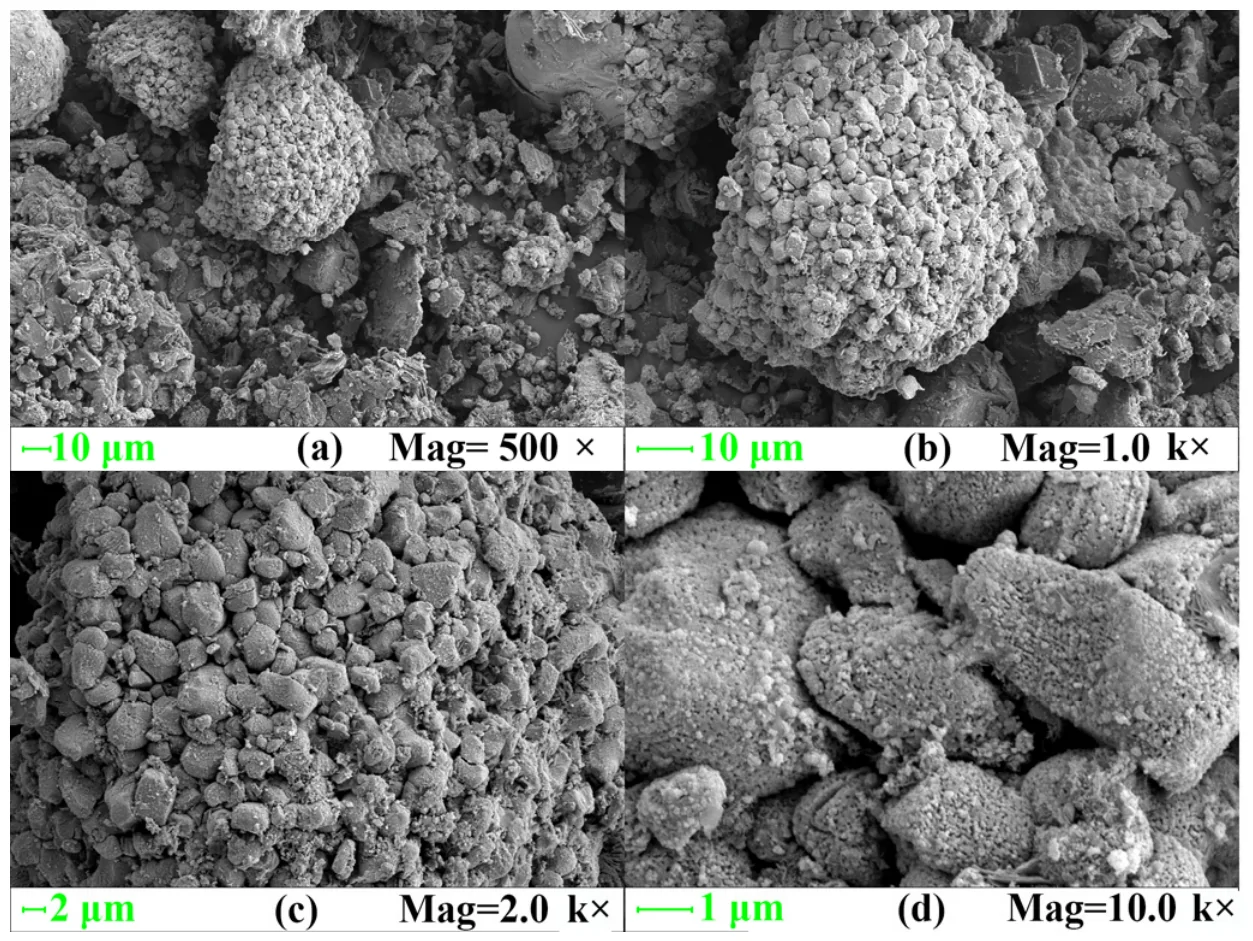
Multiscale SEM images of the composite solid ejecta recovered from three repeated nitrogen-atmosphere thermal-runaway tests.

**Figure 4:**
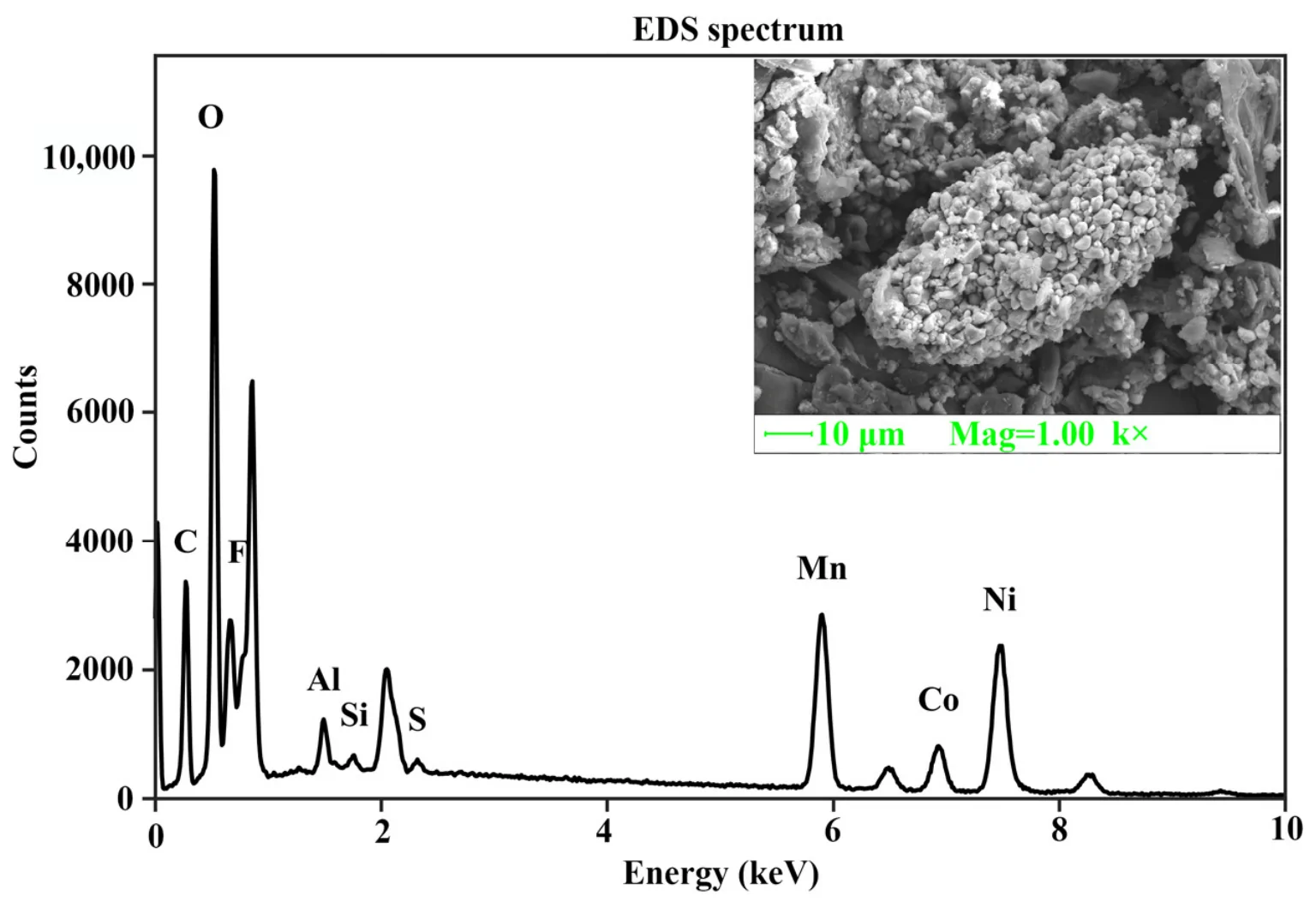
Local EDS characterization of a representative solid-ejecta region. The inset SEM image indicates the selected EDS analysis area.

**Figure 5:**
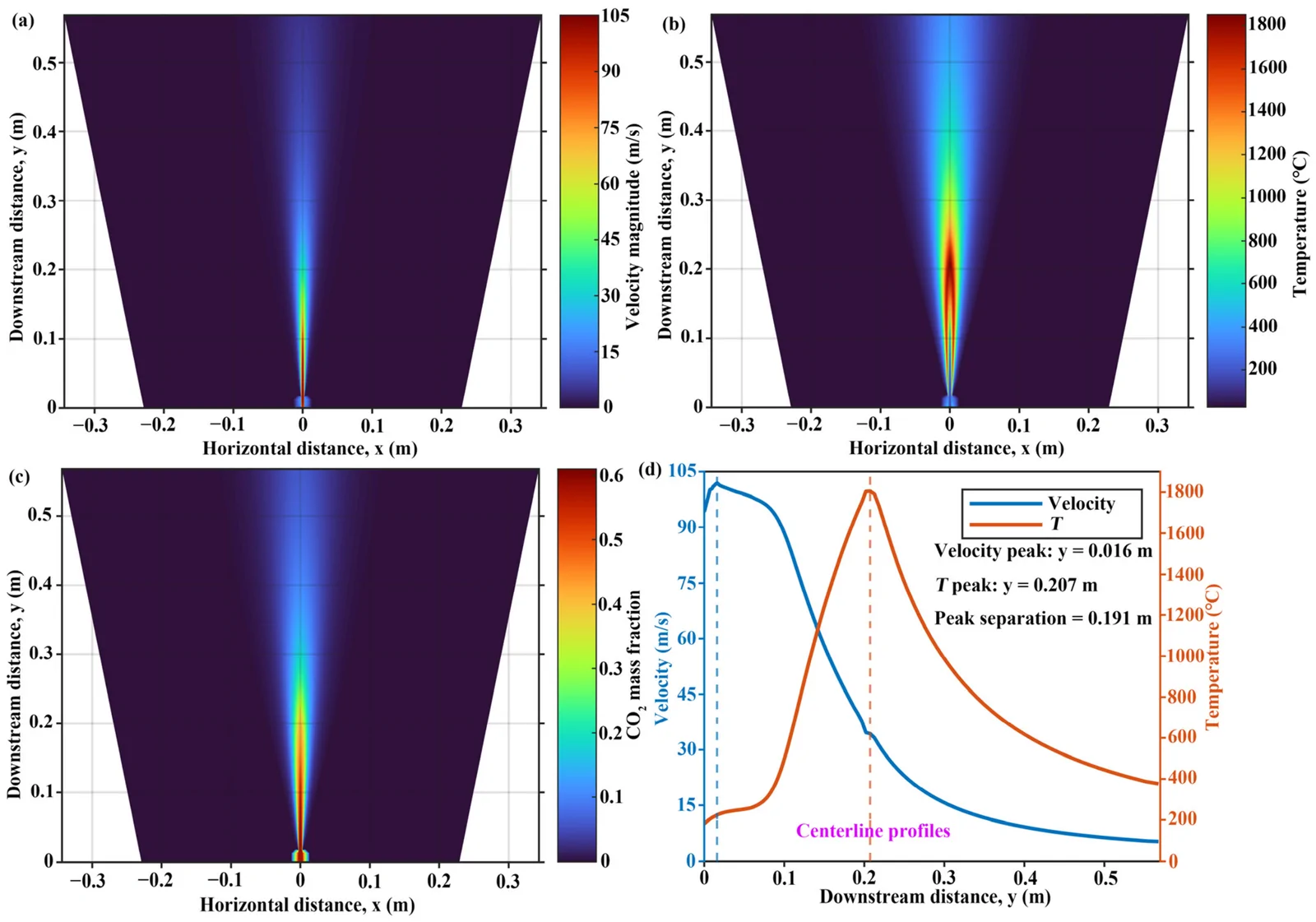
Representative near-field thermal-flow and CO_2_ transport results under the high-intensity ejection condition: (**a**) velocity-magnitude field, (**b**) temperature field, (**c**) CO_2_ mass-fraction field, and (**d**) centerline velocity and temperature profiles.

**Figure 6:**
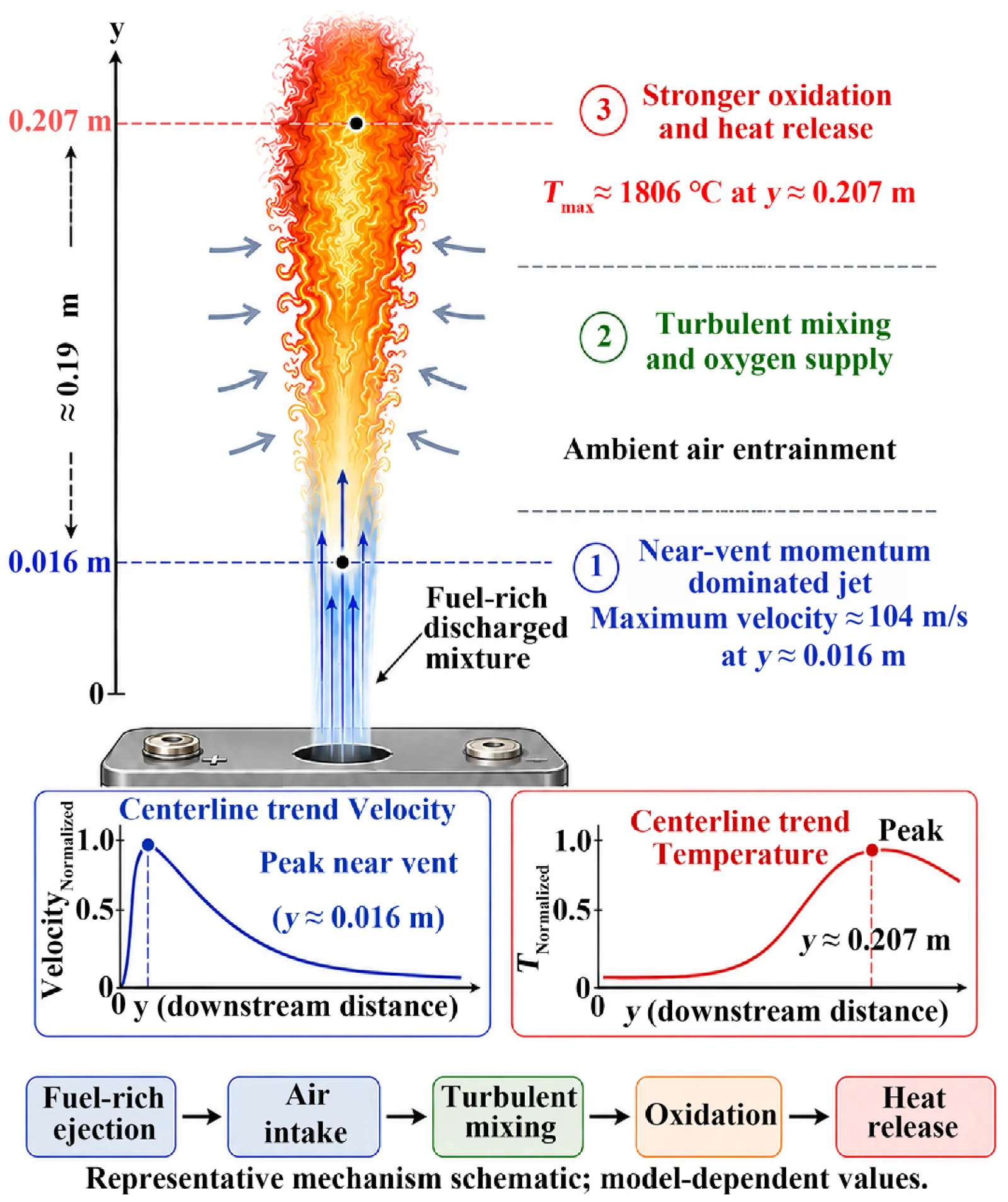
Schematic mechanism of near-vent high-velocity ejection and downstream heat release in a thermal-runaway reacting jet.

**Table 1:** Summary of cell characteristics, material characterization, and representative numerical conditions.

Category	Parameter	Value
Cell	Nominal capacity	58 Ah
Cell	Cathode chemistry	NCM811
Cell	Anode	Graphite
Cell	Cell mass	926 ± 15 g
Cell	Dimensions	148.24 × 26.66 × 102.8 mm
Cell	State of charge (SOC)	100%
Solid ejecta	Sample origin	Equal-mass composite sample pooled from three repeated 0% O_2_ (N_2_) thermal-runaway tests
SEM/EDS	Instrument	Merlin Compact field-emission SEM with EDS (Carl Zeiss Microscopy GmbH, Jena, Germany)
SEM	Magnifications	500×, 1000×, 2000×, 10,000×
EDS	Accelerating voltage	15 kV
EDS	Live acquisition time	≈60 s
Reduced model input	CO_2_	31.75 vol.%
Reduced model input	CO	35.82 vol.%
Reduced model input	H_2_	32.06 vol.%
Reduced model input	O_2_	0.37 vol.%
Numerical model	Software	COMSOL Multiphysics 6.2
Numerical model	Dimensionality	2D
Numerical model	Turbulence model	*k*-*ω*
Numerical model	Combustion treatment	Global reactions + eddy-dissipation model
Numerical model	Vent inlet width (2D model)	12.5 mm
Numerical model	Inlet velocity	90 m/s
Numerical model	Inlet-velocity basis	Reference to previously observed maximum apparent ejecta velocity
Numerical model	Inlet temperature	941.2 °C
Numerical model	Coflow air velocity	0.2 m/s
Numerical model	Ambient temperature	25 °C
Numerical model	Ambient pressure	101.325 kPa
Numerical model	Combustion regime	Non-premixed turbulent reacting jet
Numerical model	Ignition location	Safety-valve outlet
Numerical model	Eddy-dissipation parameter *α*	4
Numerical model	Eddy-dissipation parameter *β*	0.5
Numerical model	Solution type	Stationary
Numerical model	Radiation	Enabled
Numerical model	Gravity/buoyancy	Neglected
Numerical model	Outer boundary	Open boundary
Mesh	Vertices	13,101
Mesh	Elements	12,800
Mesh	Minimum element quality	0.3197
Mesh	Average element quality	0.8019
Computational domain	Area	0.1624 m^2^
Numerical outputs	Variables	Velocity, temperature, CO_2_ mass fraction

**Table 2:** Local EDS composition and possible material origins of the analyzed ejecta region.

Element	wt.%	at.%	Possible Material Origin(s)
C	15.49	32.50	Possible carbonaceous/graphite source; contribution from sample preparation cannot be excluded
O	18.51	29.16	Possible oxygen-containing electrode residue
F	9.85	13.06	Possible electrolyte/binder residue
Al	0.91	0.85	Possible Al current-collector/structural source
Si	0.26	0.24	Trace constituent
S	0.36	0.28	Trace constituent
Mn	16.24	7.45	Possible NCM cathode contribution
Co	7.36	3.15	Possible NCM cathode contribution
Ni	31.01	13.31	Possible NCM cathode contribution

Note: The values represent a selected local EDS region and should not be interpreted as the bulk-average composition of the entire ejecta sample.

**Table 3:** Selected comparison of reported elemental compositions of LIB TR particles.

Study	Battery/Test and Sampling Basis	Reported Elemental Composition (wt.%)	Comparison Note
Present study	58 Ah NCM811; 100% SOC; N_2_; settleable solids brushed from chamber bottom after cooling; equal-mass pooled sample; local EDS ROI	C 15.49; O 18.51; F 9.85; Al 0.91; Mn 16.24; Co 7.36; Ni 31.01; Ni + Mn + Co 54.61	Local EDS region; not a bulk-average ejecta composition
Yang et al. (2021) [26]	NMC811 thermal-runaway particles	Ni + Mn + Co 77.59; Cu 3.09; Al 12.3	Same nominal cathode chemistry, but sampling and reporting basis are not identical to the present local ROI
Zhang et al. (2019), summarized in [28]	50 Ah NMC311 prismatic cell; 100% SOC; air; settleable particles	C 30; Ni 20; Mn 6; Co 6; Cu 8	Different cathode composition and oxygen-containing atmosphere
Zhang et al. (2019), summarized in [28]	50 Ah NMC311 prismatic cell; 100% SOC; N_2_; settleable particles	C 28.0484; Ni 20.7536; Cu 9.4675; Al 9.3793; Co 6.9576; Li 3.6400	Same inert atmosphere, but different cathode chemistry and analytical/reporting basis
Wang et al. (2021), summarized in [29]	50 Ah NMC311 prismatic cell; 97% SOC; N_2_; particle-size-fractionated samples	Ni ~40; Cu ~15; Co ~11; Al 11–40; Li 1–11	Element fractions varied with particle size, illustrating sampling dependence

Note: Literature percentages are presented as reported or summarized in Li et al. [27]. Because particle-size range, collection atmosphere/location, analytical method, and normalization basis differ among studies, the values should not be interpreted as directly equivalent measurements. “Local EDS ROI” denotes the selected region of interest analyzed in the present study.

## Data Availability

The original contributions presented in this study are included in the article. Further inquiries can be directed to the corresponding author.

## Abbreviations

The following abbreviations are used in this manuscript:

TR	Thermal runaway
LIB	Lithium-ion batteries
NCM811	Li(Ni_0.8_Co_0.1_Mn_0.1_)O_2_
*T*_E_	Peak jet-zone temperature
*T*	Temperature
SEM	Scanning electron microscopy
EDS	Energy-dispersive X-ray spectroscopy
SOC	State of charge
